# Urinary Liver‐Type Fatty Acid‐Binding Protein, Albuminuria, and eGFR Trajectories in Type 2 Diabetes

**DOI:** 10.1111/1753-0407.70285

**Published:** 2026-10-16

**Authors:** Hiroaki Satoh, Shotaro Nakamura, Ayako Haba, Naoki Sekine, Daisuke Sugimoto

**Affiliations:** ^1^ Department of Diabetes and Endocrinology Juntendo University Urayasu Hospital Urayasu Chiba Japan

**Keywords:** albuminuria, diabetic kidney disease, eGFR trajectory, Type 2 diabetes, urinary liver‐type fatty acid‐binding protein

## Abstract

**Background:**

Urinary liver‐type fatty acid‐binding protein (L‐FABP) reflects proximal tubular stress and has been proposed as a biomarker of diabetic kidney disease. We examined whether urinary L‐FABP provides information on longitudinal kidney function beyond urinary albumin‐to‐creatinine ratio (UACR).

**Methods:**

This retrospective cohort study included patients with type 2 diabetes who had UACR and urinary L‐FABP measured simultaneously at the initial visit and underwent outpatient follow‐up. The analysis included patients with at least three eGFR measurements over at least 2 years. eGFR trajectories were evaluated using linear mixed‐effects models with random intercepts and slopes. UACR and L‐FABP were analyzed as log(1 + *x*)‐transformed continuous variables.

**Results:**

The analysis included 1,031 patients (age 58.1 years; 65.1% men), with a median follow‐up of 4.76 years and a median of 10 eGFR measurements. Urinary L‐FABP correlated with UACR (Spearman *ρ* = 0.526; *p* < 0.001). Both biomarkers were independently associated with lower baseline eGFR. In a model without UACR, higher L‐FABP was associated with faster eGFR decline (−0.98 mL/min/1.73 m²/year per unit increase in log[1 + L‐FABP]; *p* < 0.001). After adjustment for continuous UACR, the association was attenuated and no longer significant (−0.19; 95% CI, −0.49 to 0.11; *p* = 0.221), whereas UACR remained independently associated with eGFR decline. Results were unchanged after further adjustment for baseline eGFR and lipid parameters.

**Conclusions:**

Urinary L‐FABP was associated with baseline kidney impairment but did not independently predict eGFR trajectory after continuous UACR was taken into account. Urinary L‐FABP reflects kidney injury severity, although its incremental prognostic value beyond quantitatively assessed albuminuria was limited in this cohort.

## Graphical abstract

Urinary L‐FABP predicted eGFR decline and persistent albuminuria before UACR adjustment; however, both associations disappeared after adjustment for continuous baseline UACR, indicating that quantitatively assessed UACR, even within the normoalbuminuric range, was the dominant prognostic marker.

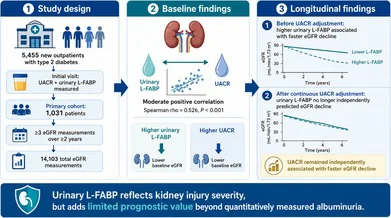

To the Editor,

Estimated glomerular filtration rate (eGFR) and urinary albumin‐to‐creatinine ratio (UACR) are the principal clinical markers for diabetic kidney disease [1]. Urinary liver‐type fatty acid‐binding protein (L‐FABP), a marker of proximal tubular stress, may become elevated before overt albuminuria and has been proposed as a predictor of diabetic nephropathy onset and progression [2, 3, 4, 5, 6]. However, L‐FABP and UACR are correlated, and it remains uncertain whether L‐FABP provides prognostic information beyond the absolute UACR level. We therefore examined whether baseline urinary L‐FABP independently predicted longitudinal eGFR decline and incident persistent albuminuria in patients with Type 2 diabetes.

## 1 Methods

This single‐center retrospective cohort study included outpatients with Type 2 diabetes who had simultaneous UACR and urinary L‐FABP measurements at their initial visit between January 2016 and September 2024. eGFR was calculated using the equation developed for the Japanese population [7]. For trajectory analysis, patients were required to have at least three eGFR measurements spanning at least 2 years, consistent with recommendations for more reliable estimation of eGFR slopes [8]. Linear mixed‐effects models with patient‐specific random intercepts and slopes were adjusted for age, sex, and glycated hemoglobin, with and without continuous baseline UACR. UACR and L‐FABP were modeled as log(1 + *x*)‐transformed continuous variables.

For incident albuminuria analysis, patients with baseline UACR < 30 mg/gCr and at least two subsequent UACR measurements were included. Persistent albuminuria was defined as two consecutive follow‐up UACR values ≥ 30 mg/gCr; the event date was the second qualifying measurement. Cox proportional hazards models assessed baseline L‐FABP, first unadjusted, then adjusted for continuous baseline UACR, and finally for age, sex, glycated hemoglobin, and baseline eGFR. Cox analyses used complete cases; missingness could not be assumed to be missing completely at random (MCAR), and no imputation or missing‐data sensitivity analysis is presented. The institutional review board approved the study and waived individual consent (E25‐0362‐U01).

## 2 Results

The eGFR analysis included 1031 patients (mean age 58.1 years; 65.1% men), with 14 103 eGFR measurements over a median 4.76 years. Urinary L‐FABP correlated with UACR (Spearman *ρ* = 0.526; *p* < 0.001; Figure 1). Higher L‐FABP was associated with faster eGFR decline before UACR adjustment (difference in annual eGFR change per unit increase in log[1 + L‐FABP], −0.98 mL/min/1.73 m^2^/year; *p* < 0.001), but not after continuous UACR adjustment (−0.19; 95% confidence interval [CI], −0.49 to 0.11; *p* = 0.221). UACR remained independently associated with eGFR decline (−0.62; 95% CI, −0.77 to −0.48; *p* < 0.001).

**FIGURE 1:**
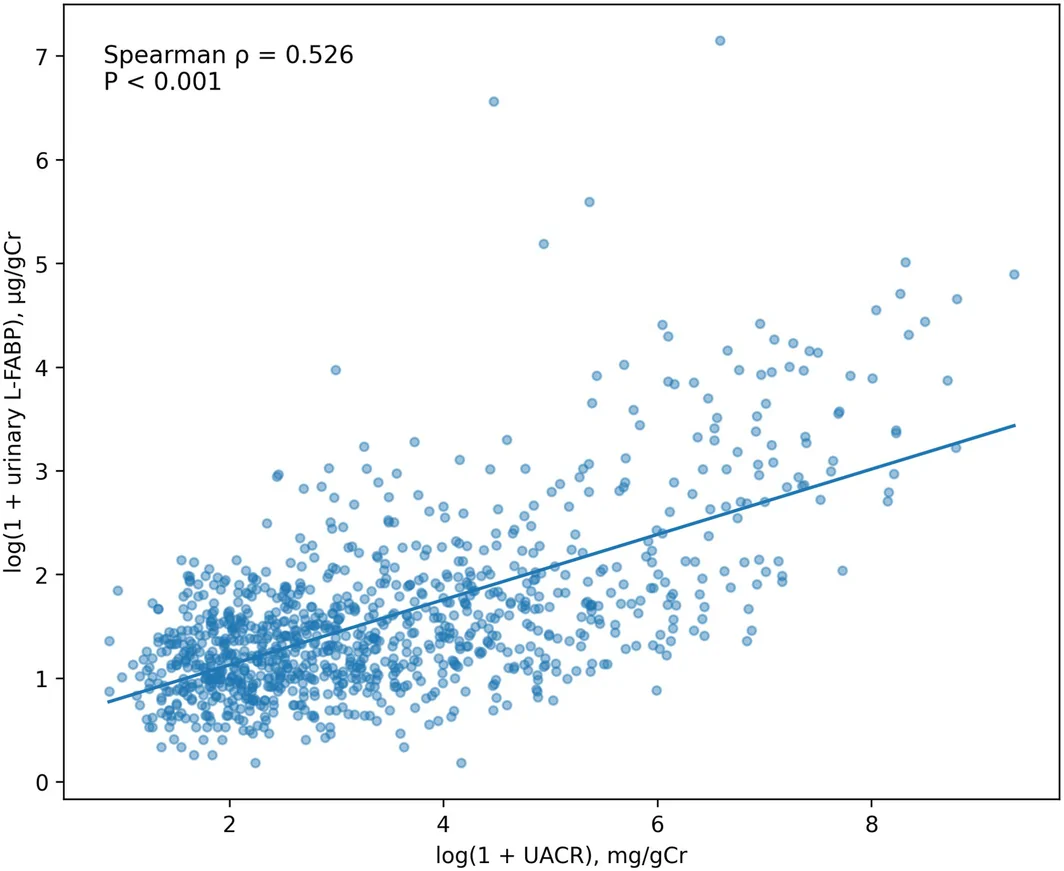
Correlation between urinary L‐FABP and UACR. Both variables are shown after log(1 + *x*) transformation. Spearman *ρ* = 0.526; *p* < 0.001. L‐FABP, liver‐type fatty acid‐binding protein; UACR, urinary albumin‐to‐creatinine ratio.

Among 1057 patients with baseline normoalbuminuria, 235 developed persistent albuminuria. Complete‐case analysis included 935 patients (208 events), excluding 122 (11.5%) with missing covariate data. In this analysis, L‐FABP was associated with persistent albuminuria before adjustment (hazard ratio [HR], 1.46 per unit increase in log[1 + L‐FABP]; 95% CI, 1.12–1.91; *p* = 0.005), but not after adjustment for baseline UACR (HR, 0.95; 95% CI, 0.72–1.26; *p* = 0.739) or full adjustment (HR, 0.87; 95% CI, 0.65–1.16; *p* = 0.333). Baseline UACR remained strongly associated with persistent albuminuria in the fully adjusted model (HR, 5.00; 95% CI, 3.78–6.61; *p* < 0.001). Results are summarized in Table 1.

**TABLE 1:** Associations of urinary L‐FABP and UACR with longitudinal kidney outcomes.

Outcome and model	Biomarker	Estimate	95% CI	*p*
Annual eGFR change: age, sex, HbA1c adjusted	L‐FABP	−0.98	−1.23 to −0.73	< 0.001
Annual eGFR change: + continuous UACR	L‐FABP	−0.19	−0.49 to 0.11	0.221
Annual eGFR change: + continuous UACR	UACR	−0.62	−0.77 to −0.48	< 0.001
Persistent albuminuria: unadjusted	L‐FABP	HR 1.46	1.12 to 1.91	0.005
Persistent albuminuria: + baseline UACR	L‐FABP	HR 0.95	0.72 to 1.26	0.739
Persistent albuminuria: fully adjusted	L‐FABP	HR 0.87	0.65 to 1.16	0.333
Persistent albuminuria: fully adjusted	UACR	HR 5.00	3.78 to 6.61	< 0.001

*Note:* Coefficients for eGFR represent the difference in annual eGFR change per one‐unit increase in the log(1 + *x*)‐transformed biomarker. The fully adjusted Cox model included baseline UACR, age, sex, HbA1c, and baseline eGFR.

Abbreviations: CI, confidence interval; HR, hazard ratio.

## 3 Comment

Urinary L‐FABP was associated with both faster eGFR decline and incident persistent albuminuria before UACR adjustment. Neither association remained statistically significant after adjustment for continuous baseline UACR, suggesting that the prognostic information captured by L‐FABP substantially overlaps with quantitatively measured albuminuria.

Previous studies suggest that L‐FABP may rise before increased albumin excretion, identify early tubular injury, and predict kidney function decline [2, 3, 4, 5, 6]. Our findings do not exclude this biological role but emphasize the importance of adjusting for continuous baseline UACR, even within the normoalbuminuric range.

Limitations include the retrospective single‐center design, nonprotocolized UACR measurement frequency, lack of blood pressure and kidney‐protective medication data, and potential informative censoring. Differential use or initiation of renin–angiotensin system inhibitors or sodium–glucose cotransporter 2 inhibitors could reduce albuminuria and slow long‐term eGFR decline [1], potentially attenuating biomarker associations if higher‐risk patients received treatment more often. Treatment‐related early eGFR changes and unmeasured blood pressure differences could also bias these associations; the net direction is uncertain. Exclusion of patients with missing covariates may introduce selection bias; the complete‐case results therefore require cautious interpretation.

In this cohort, baseline L‐FABP was not independently associated with either outcome after continuous UACR adjustment. Serial L‐FABP measurements and evaluation in high‐risk groups, such as patients with rapid eGFR decline, may still have clinical value; prospective studies incorporating hard kidney outcomes are needed to establish their incremental utility.

## Author Contributions

Hiroaki Satoh conceived and designed the study, interpreted the data, and drafted the manuscript. Shotaro Nakamura performed the statistical analyses and contributed to data interpretation. Shotaro Nakamura, Ayako Haba, Naoki Sekine, and Daisuke Sugimoto contributed to data acquisition and critically revised the manuscript. All authors approved the final version of the manuscript and agree to be accountable for all aspects of the work.

## Funding

This work was supported by JSPS KAKENHI Grant Number JP23K10969 (H.S.).

## Conflicts of Interest

The authors declare no conflicts of interest.

## Data Availability

Anonymized datasets that support the findings of this study are available from the corresponding author upon reasonable request.
